# ﻿A new species of the jawfish genus *Opistognathus* from Taiwan, northwestern Pacific Ocean (Perciformes, Opistognathidae)

**DOI:** 10.3897/zookeys.1220.123541

**Published:** 2024-12-09

**Authors:** Yo Su, Hsuan-Ching Ho

**Affiliations:** 1 Department and Graduate Institute of Aquaculture, National Kaohsiung University of Science Technology, Kaohsiung, Taiwan National Kaohsiung University of Science Technology Kaohsiung Taiwan; 2 Institute of Marine Biology, National Dong Hwa University, Pingtung, Taiwan National Dong Hwa University Pingtung Taiwan; 3 Australian Museum, Sydney, Australia Australian Museum Sydney Australia

**Keywords:** Actinopterygii, biodiversity, ichthyology, morphology, taxonomy

## Abstract

A new species of jawfish genus *Opistognathus* is described based on a specimen collected from a beach in the Peng-hu Islands during a cold snap. The new species, *Opistognathuscryos***sp. nov.**, differs from its congeners in having a rigid upper jaw, 10–11 + 1 + 19–22 = 31–33 gill rakers, 55 scale rows in lateral series, 10 + 16 = 26 vertebrae, the terminus of the lateral line at the base of the fourth segmented dorsal-fin ray (15^th^ in total rays), the head, nape, dorsal-fin base above lateral line, throat, chest, and pectoral-fin base naked, dorsal fin with eight blotches along its entire base, body with five horizontal dark stripes, nape with two dark blotches in front of the dorsal-fin origin, and a caudal fin with five narrow, dark bands. A detailed description is provided and compared to its similar congeners.

## ﻿Introduction

The jawfish family Opistognathidae is a group of small to moderately sized fishes (up to 50 cm, but mostly less than 12 cm) that are distributed circumglobally, except for the Mediterranean Sea and the eastern Atlantic Ocean (Smith-Vaniz 1999, 2023). They are well known for their moth-brooding behavior, in which parents carry their sticky egg mass in their mouth until hatching (Smith-Vaniz 1999). They are bottom burrowers that inhabit sandy bottoms at depths of 2–30 m, with some Indo-West Pacific species down to 200 m (Smith-Vaniz 1999), and Caribbean species to at least 300 m (Smith-Vaniz 2017).

Currently, four genera are recognized as valid: *Anoptoplacus* Smith-Vaniz, 2017, *Lonchopisthus* Gill, 1862, *Opistognathus* Cuvier, 1816, and *Stalix* Jordan & Snyder, 1902. Among the four genera, *Opistognathus* can be discriminated from other genera in having the anterior dorsal-fin spines not transversely forked; the posterior margin of upper jaw straight or rounded; dorsal-fin rays, X–XIII, 10–22; anal-fin rays II–III, 10–20; the caudal fin rounded, with 17–18 principal rays; and the infraorbital bones not plate-like (Smith-Vaniz 1999, 2003, 2017).

A recent revision of Indo-West Pacific *Opistognathus* by Smith-Vaniz (2023) recognized 60 species, including 18 new species. Moreover, he provided diagnostic characters and an identification key for all Indo-West Pacific species, and he noted that more undescribed species occur in this region. Thereafter, Fujiwara et al. (2023) and Fujiwara and Ikeda (2024) described *Opistognathusctenion* Fujiwara, Shinohara & Motomura, 2023 and *Opistognathusabei* Fujiwara & Ikeda, 2024, respectively. Therefore, the total number of *Opistognathus* is 93, including 62 from the Indo-West Pacific, 17 from the western Atlantic, and 14 from the eastern Pacific (Smith-Vaniz 2023; Fujiwara and Ikeda 2024).

Penghu is a group of small islands in the Taiwan Strait off western Taiwan, with the Tropic of Cancer passing through it. In the historical records, several serious cold-related incidents have occurred along the northern coast during the winter seasons in Penghu, such as those in 1977, 2008, and 2022. The cold fronts brought low-temperature longshore currents from the north, causing the water temperature to drop below 14 °C. Consequently, many coral-reef fishes were frozen to death and washed ashore (Hsieh et al. 2012; Ho pers. observ.).

During the cold snap in 2022, many stranded fishes were collected and sent back to the lab. Among them, an apparently unnamed species of *Opistognathus* with a color pattern closely similar to *Opistognathus* sp. 1 of Smith-Vaniz (2023: fig. 103). The unnamed jawfish is here described as *O.cryos* and differs from other sympatric congeners in having a unique coloration and several morphological characters. A detailed description of the new species is provided and compared to its congeners.

## ﻿Materials and methods

The specimen was fixed in 4% formaldehyde and subsequently transferred to 70% ethanol for preservation in the Pisces Collection of the National Museum of Marine Biology and Aquarium, Pingtung, Taiwan (**NMMB-P**).

Terminology and methodology follow Smith-Vaniz (2023). Medial fin rays, vertebral formula, and both dorsal- and anal-fin interdigitation patterns were determined using X-radiographs taken by a digital X-ray machine set up in the National Museum of Marine Biology and Aquarium. The supraneural bones were abbreviated as “S” in the fin interdigitation patterns. Paired characters were expressed as left/right whenever available. Head pores were observed on both sides directly under a stereomicroscope (Olympus SZ51), with partial drying and adjusting light directions to enhance the detection. Illustrations of head pores were traced from magnified photographs using Adobe Photoshop.

Measurements were taken from the right side of the specimen using digital calipers rounding to the nearest 0.1 mm under a stereomicroscope. Morphometric data were expressed as ratios or percentages of standard length (**SL**) and head length (**HL**), except where otherwise indicated.

## ﻿Results

**Family Opistognathidae**

### ﻿ Opistognathus cryos sp. nov.

Taxon classificationAnimaliaPerciformesOpistognathidae

13BF485C-3ACE-583C-B902-03D6804ACF4B

https://zoobank.org/8F808D49-8B17-4E9C-A4B8-E273BAC2742C

**Figure 1.**
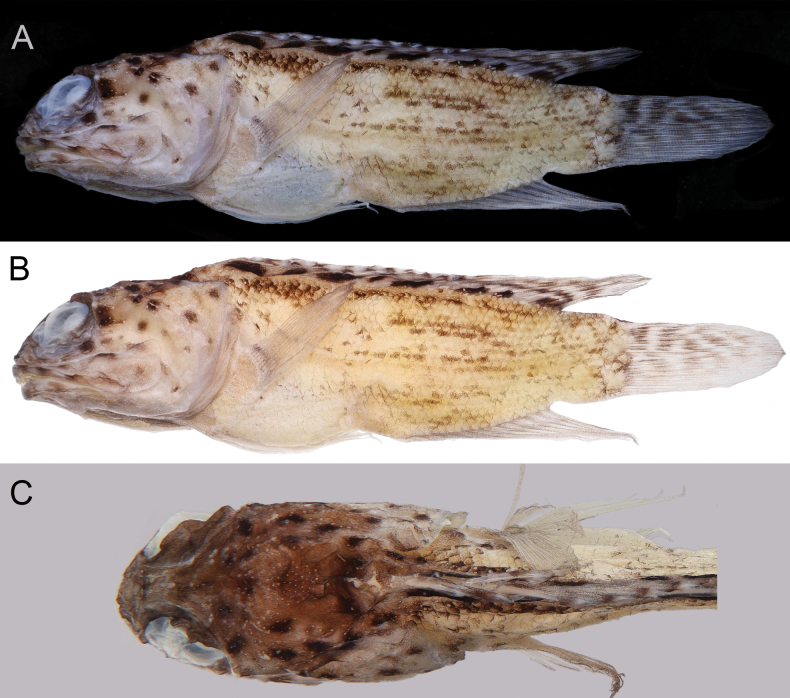
Preserved specimen of *Opistognathuscryos* sp. nov., holotype, NMMB-P 36179, 65.1 mm SL**A** on black background **B** on white background **C** dorsal view of nape. Photos by Y.-C. Hsu.

**Figure 2.**
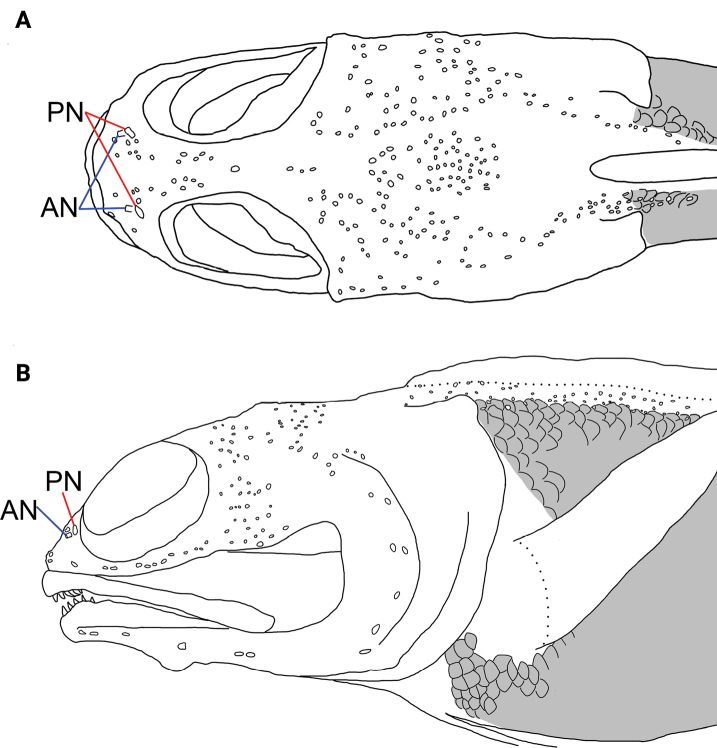
Head pores and adjacent area of *Opistognathuscryos* sp. nov., holotype, NMMB-P 36179, 65.1 mm SL, with scaled area shaded in gray **A** dorsal view **B** lateral view. Abbreviations: AN = anterior nostril; PN = posterior nostril. Dotted lines indicate dorsal- and pectoral-fin bases. Anterior to left. Figure not to scale.

**Figure 3.**
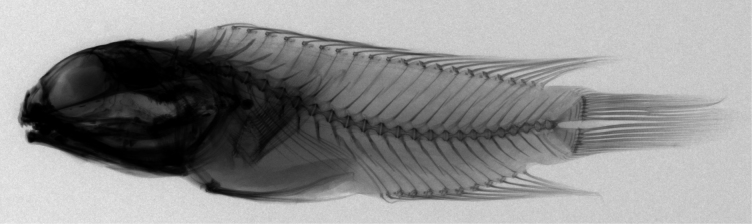
X-radiograph of *Opistognathuscryos* sp. nov., holotype, NMMB-P 36179, 65.1 mm SL.

Figs 1 , 2 , 3 , Tables 1 , 2 New English name: Frozen jawfish New Chinese name: 冷鋒後頷鱚

Opistognathus sp. 1: Smith-Vaniz 2023: 72, fig. 103.

#### Type locality.

Taiwan, Peng-hu Islands, Chih-kan beach, ca 23°40'12"N, 119°36'10"E, 25 February 2022, H.-C. Ho leg.

#### Type specimen.

Holotype: NMMB-P 36179, 65.1 mm SL.

#### Diagnosis.

A species of *Opistognathus* differing from its congeners in having the following combination of characters: upper jaw rigid, without a distinct flexible lamina posteriorly; dorsal-fin rays XI, 11; anal-fin rays II, 10; gill rakers 10–11 + 1 + 19–22 = 31–33; scale rows in lateral series 55; vertebral formula 10 + 16 = 26; dorsal-fin interdigitation pattern S/S/1/1+1/1/; lateral-line ends at base of fourth segmented dorsal-fin ray (15^th^ in total rays); head, nape, dorsal-fin base above lateral line, throat, chest, and pectoral-fin base scaleless; upper two preopercular and fifth mandibular pores bipored; vomer edentate; head mottled with small, dark blotches; dorsal fin with eight blotches along its base; distal portion of membrane between dorsal-fin spines white; body with five horizontal, dark stripes; nape with two dark blotches in front of dorsal-fin origin; and caudal fin with five narrow, dark bands.

#### Description.

Meristic and morphometric data are provided in Tables 1, 2. Paired characters are presented as left/right whenever available.

**Table 1.** Meristic characters of *Opistognathuscryos* sp. nov. and similar species. Paired characters are presented as left/right whenever available. Data of other species were retrieved from Smith-Vaniz (2023).

	*O.cryos* sp. nov.	*O.asper*	*O.liturus*
	This study	Smith-Vaniz (2023)	Smith-Vaniz (2023)
	Holotype	All types (*n* = 3)	Holotype
Dorsal-fin rays	XI, 11	XI, 11–12	XI, 11
Pectoral-fin rays	19/19	19	19
Anal-fin rays	II, 10	II, 10	II, 10
Gill rakers	11 + 1+ 19 = 31/10 + 1 + 22 = 33	10–11 + 22–24 = 32–34	9 + 1 + 21 = 31
Scale rows in lateral series	55/55	42–44	44
Vertebrae	10 + 16 = 26	10 + 16 = 26	10 + 16 = 26
Dorsal-fin interdigitation anterior patterns	S/S/1/1+1/1/	S/S/1/1+1/1/	/ /1/1+1/1/
Lateral-line terminus total dorsal-fin rays	15/15	11–15	15

Dorsal-fin rays XI, 11. Anal-fin rays II, 10. Pectoral-fin rays 19/19. Pelvic-fin rays I, 5/I, 5. Principal caudal-fin rays 8 + 8, with innermost 6 + 6 rays branched; procurrent caudal-fin rays 4 on both upper and lower lobes. Gill rakers 11 + 1 + 19 = 31/10 + 1 + 22 = 33. Scale rows in lateral series 55/55. Vertebrae 10+16=26. Dorsal-fin interdigitation pattern S/S/1/1+1/1/; anal-fin interdigitation pattern /1/1/1/1/1/.

Body slender, depth at anal-fin origin 4.2 in SL; both dorsal and ventral profiles of body flat. Head large, length 2.5 in SL; anterior profile of head rounded, gently curved to dorsal-fin origin. Eyes large, eye diameter 3.0 in HL. Two nostrils, anterior one a short tube with small flap; posterior one without flap, situated immediately in front of eye. Preoprecle and opercle covered by skin, their posterior margins without spines; single, small flap present on upper end of opercle.

Mouth lower in position, slightly oblique, forming ca 10° angle with body axis. Jaws terminal, with lower jaw slightly included. Upper-jaw length 1.5 in HL, its end exceeding 0.8 in eye diameter behind posterior margin of orbit; posterior margin of maxilla truncated, without distinct flexible lamina; its anterior margin without crenulae. Supramaxilla present, greatest width about one-third of maxilla. Premaxilla with two or three rows of small canine teeth anteriorly; posterior portion forming single row of conical teeth, their size decreasing posteriorly. Dentary with small canine teeth forming two or three rows anteriorly and single row posteriorly. Vomer and palatine without teeth.

Body scales cycloid. Scales absent on head, nape, dorsal-fin base above lateral line, throat, chest, and pectoral-fin base (Fig. 2). Lateral-line terminus below 4^th^/4^th^ or 15^th^/15^th^ in soft ray or total elements of dorsal fin, respectively; lateral-line pores arranged in irregular series along embedded lateral-line canals. Cephalic sensory pores rather sparsely scattered (Fig. 2); upper-two preopercular pores bipored, others simple; fifth mandibular pore bipored, others simple.

Dorsal fin with long base, originated at vertical through upper end of gill slit; its distal nearly straight, with slight elevation on soft rays; no distinct notch between spines and soft rays. Pectoral-fin tip pointed; its origin at same horizontal through lower margin of eye; its tip reaching near vertical through anal-fin origin. Pelvic fin elongated, its origin below second dorsal-fin spine and in advance of pectoral fin; its tip reaching anal-fin origin when adpressed; outermost segmented ray not bounded to adjacent ray, with incision on interradial membrane. Anal-fin base moderately long, its posterior end slightly anterior to that of dorsal fin; its origin below first and second dorsal-fin soft rays (between 12^th^ and 13^th^ total dorsal-fin rays); fin rays gradually longer posteriorly; its distal margin nearly straight. Caudal fin rounded, slightly pointed; its length 1.4 in HL. Caudal peduncle broad, depth 2.8 in HL.

#### Coloration.

When preserved (Fig. 1A, B), body pale, with one horizontal dark line near dorsal-fin base, and four rows of thinner dark lines on lateral side of body above anal-fin base. Head pale, scattered with irregularly sized dark blotches on operculum, upper and lower jaws, and infraorbital; their sizes smaller than pupil. Nape with two dark blotches in front of dorsal-fin origin (Fig. 1C). Dorsal fin dusky, with eight dark blotches along its base, slightly extending onto dorsum; distal end of fin spines white; soft rays with two horizontal, dark stripes. Caudal fin dusky, with five narrow, dark bands. Anal, pectoral, and pelvic fins dusky. Oral cavity, including underside of tongue pale.

#### Osteology.

Pleural ribs present on fourth to tenth vertebra; epineurals present and epipleurals absent. Caudal skeleton consists of four plates, including three hypurals and one parhypural; hypural 1 and 2 fused; hypural 3 and 4 fused; hypural 5 present (Fig. 3).

#### Etymology.

The specific name *cryos*, is from the Greek “κρύος” meaning cold or chilled, indicating that the holotype was collected during a cold snap in 2022. The common name “frozen jawfish” is also a reference to the fantasy film “Frozen” produced by Walt Disney Animation Studios.

#### Distribution.

The holotype was collected ashore from the northern portion of the Peng-hu Islands, western Pacific Ocean. Another possible record from Japan (Smith-Vaniz 2023) may suggest a wide distribution in the northwestern Pacific Ocean.

## ﻿Discussion

According to the key provided by Smith-Vaniz (2023), *Opistognathuscryos* sp. nov. mostly resembles *O.asper* Smith-Vaniz, 2023, a deep-water species from northwestern Australia; both species share a rigid upper jaw, its end without a flexible lamina, dark blotches on the dorsum extending onto the dorsal fin, and similar meristic counts. However, *O.cryos* can be discriminated from *O.asper* in having scales rows in horizontal series 55 (vs 42–44 in *O.asper*; Table 1); the upper two preopercular pore bipored and others simple (vs all bipored); the pectoral-fin base naked (vs scaly); eight dark blotches along the dorsal-fin base (vs 4–5); and nape with two dark blotches in front of the dorsal fin (Fig. 1C; blotch absent). Other differences in morphometric data (Table 2) need further study since the holotype of *O.cryos* is larger than all specimens of *O.asper* (65.1 mm SL vs 34.4–50.2 mm SL in *O.asper*).

**Table 2.** Morphometric characters of *Opistognathuscryos* sp. nov. and similar species. Data of other species were retrieved from Smith-Vaniz (2023). Abbreviations: A = anal-fin; C = caudal-fin; D = dorsal-fin; HL = head length; SL = standard length; V = pelvic-fin.

	*O.cryos* sp. nov.	*O.asper*	*O.liturus*
	This study	Smith-Vaniz (2023)	Smith-Vaniz (2023)
	Holotype	All types (*n* = 3)	Holotype
SL (mm)	65.1	34.4–50.2	55.3
%SL			
HL	39.5	37.0–40.3	37.8
Body depth at A origin	23.9	23.7–25.6	25.0
Postorbital length	23.5	20.6–21.4	21.9
Eye diameter	13.2	12.3–13.5	12.3
Upper-jaw length	26.3	24.3–26.2	26.2
Postorbital-jaw length	10.8	–	8.7
Predorsal length	35.7	36.9–40.8	35.8
Preanal length	62.3	61.8–66.9	63.3
Length of D base	57.5	60.0–64.2	59.7
Length of A base	24.9	23.3–27.8	27.3
V length	32.4	29.3–35.2	31.1
C length	28.9	30.2–43.0	30.6
Caudal-peduncle depth	14.1	13.5–15.1	14.8
%HL			
Postorbital length	59.4	52.7–57.4	57.9
Eye diameter	33.4	33.5–35.1	32.5
Upper-jaw length	66.6	65.0–67.7	69.4
Postorbital-jaw length	27.4	23.8–26.2	23.1

*Opistognathuscryos* is also similar to *O.liturus* Smith-Vaniz & Yoshino, 1985, both occurring in the northwestern Pacific Ocean. However, *O.cryos* differs from the latter in having scale rows in horizontal series 55 (vs 44 in *O.liturus*; Table 1), dorsal-fin interdigitation anterior pattern S/S/1/1+1/1/ (vs / /1/1+1/1/), eight dark blotches along the entire dorsal-fin base (vs four blotches extending slightly onto fin base and terminated at middle); and the fifth mandibular pore bipored (vs simple).

Moreover, the combination of gill rakers 10–11 + 1 + 19–22 = 31–33, scale rows in lateral series 55, mottled head, and eight dark blotches along the dorsal-fin base readily distinguishes it from other Indo-Pacific congeners, including *O.ctenion* and *O.abei*, which were described subsequent to Smith-Vaniz (2023) (Fujiwara et al. 2023; Fujiwara and Ikeda 2024).

### ﻿Species diversity of jawfishes in Taiwan

Table 3 listed species of jawfishes recorded from Taiwan historically, as well as the current museum specimens deposited in NMMB-P. Twelve species and two genera were recognized. Among them, *O.cryos* sp. nov., *O.flavidus* Smith-Vaniz, 2023, *O.microspilus* Smith-Vaniz, 2023, *O.variabilis* Smith-Vaniz, 2009, and *Stalixsheni* Smith-Vaniz, 1989 were described based on specimens collected from Taiwan. Moreover, the records of four species–*O.macrolepis* Peters, 1866, *O.microspilus*, and *O.solorensis* Bleeker, 1853–were based on a single voucher specimen from Taiwan.

**Table 3.** Checklist of opistognathids recorded from Taiwan.

Species	Chinese name	Reference	Remarks
*Opistognathuscastelnaui* Bleeker, 1859	卡氏後頷鱚	Mok 1993; Chen et al. 2010; Shen and Wu 2011; Tashiro 2019; Smith-Vaniz 2023	NMMB-P specimens: A total of 16 lots of 29 specimens.
*O.cryos* Su & Ho sp. nov.	冷峰後頷鱚	This study	
*O.evermanni* (Jordan & Snyder, 1902)	艾氏後頷鱚	Mok 1993; Shen and Wu 2011; Smith-Vaniz 2023	
*O.flavidus* Smith-Vaniz, 2023	金鰭後頷鱚	Shen et al. 1986; Mok 1993; Smith-Vaniz 2023	Three paratypes (NMMB-P35987, NTUM 6177 and 7112) were collected from Taiwan. Shen et al. (1986) reported as *O.fasciatus* and Mok (1993) reported as *O.evermanni*. NMMB-P specimens: NMMB-P 38449.
*O.hongkongiensis* Chan, 1968	香港後頷鱚	Mok 1993; Shen and Wu 2011; Tashiro 2019; Smith-Vaniz 2023	NMMB-P specimens: NMMB-P 4071; NMMB-P 11582; NMMB-P 23855; NMMB-P 40030.
*O.hopkinsi* (Jordan & Snyder, 1902)	霍氏後頷鱚	Smith-Vaniz 2023	Based on a specimen (ASIZP 61792) collected from northeastern Taiwan. NMMB-P specimens: NMMB-P 32936; NMMB-P 33923; NMMB-P34432.
*O.macrolepis* Peters, 1866	大鱗後頷鱚	Smith-Vaniz 2023	Based on a specimen (NTUM 15216) purchased at market of Taiwan and without precise locality.
*O.microspilus* Smith-Vaniz, 2023	小斑後頷鱚	Smith-Vaniz 2023	Only known from the holotype (NMMB-P13933) collected from Taiwan.
*O.solorensis* Bleeker, 1853	索洛後頷鱚	Smith-Vaniz 2016, 2023	Based on a specimen (SAIAB 27653) collected from Kenting National Park.
*O.variabilis* Smith-Vaniz, 2009	多彩後頷鱚	Smith-Vaniz 2009, 2023; Chen et al. 2010	Two paratypes (ASIZP 56989) were collected from Taiwan.
*O.wass*i Smith-Vaniz, 2023	瓦氏後頷鱚	Smith-Vaniz 2023	One paratype (ASIZP 56990) was collected from Spratly Islands, South China Sea.
*Stalixsheni* Smith-Vaniz, 1989	沈氏叉棘鱚	Smith-Vaniz 1989; Mok 1993; Shen and Wu 2011	Originally described from Taiwan.

## Supplementary Material

XML Treatment for Opistognathus cryos

## ﻿Additional information

### Author ORCIDs

Yo Su https://orcid.org/0000-0002-3576-9229

Hsuan-Ching Ho https://orcid.org/0000-0003-1154-601X

### Data availability

All of the data that support the findings of this study are available in the main text.

## References

[B1] Bleeker P (1853) Bijdrage tot de kennis der ichthyologische fauna van Solor. Natuurkundige Tijdschrift voor NederlandschIndië 5: 67–96.

[B2] Bleeker P (1859) Visschen van Singapore, verzameld door Graaf Fr. De Castelnau. Natuurkundige Tijdschrift voor Nederlandsch-Indië 20(2): 236–239.

[B3] Chan WL (1968) *Opistognathushongkongiensis*, a replacement name for the jawfish *Opisthognathusfasciatus* Chan. Copeia 1968(1): 198. 10.2307/1441582

[B4] Chen J-P Shao K-T Jan R-Q Kuo J-W Chen J-Y (2010) Marine Fishes of Kenting National Park. First Revised Edition. Kenting National Park Headquarters, Kenting, 650 pp. [In Chinese]

[B5] Cuvier G (1816) Le Regne Animal. Vol. 2. s. n., Paris, 532 pp.

[B6] Fujiwara K Ikeda Y (2024) Description of a new species of *Opistognathus* (Perciformes: Opistognathidae) from the southern Japan Sea. Ichthyological Research, 1–8. 10.1007/s10228-024-00951-7

[B7] Fujiwara K Motomura H Shinohara G (2023) *Opistognathusctenion* (Perciformes, Opistognathidae): A new jawfish from southern Japan. ZooKeys 1179: 353–364. 10.3897/zookeys.1179.109813 37745622 PMC10514694

[B8] Gill TN (1862) Remarks on the relations of the genera and other groups of Cuban fishes. Proceedings. Academy of Natural Sciences of Philadelphia 14: 235–242.

[B9] Hsieh H-Y Chen K-S Tseng J-T Hsian Y-L Tsai W-S (2012) Choice of winter sea areas of aquaculture fishery in response to cold damage in Penghu. Special Bulletin of Fishery Research Institute 40: 4–8. [In Chinese]

[B10] Jordan DS Snyder JO (1902) A review of the trachinoid fishes and their supposed allies found in the waters of Japan. Proceedings of the United States National Museum 24(1263): 461–497. 10.5479/si.00963801.24-1263.461

[B11] Mok H-K (1993) Family Opistognathidae. In: Shen S-C Chen C-H Lee S-C Shao K-T Mok H-K Tseng C-S (Ed.) Fishes of Taiwan.National Taiwan University, Taipei, 478–479. [pls 161-6–161-9] [In Chinese]

[B12] Peters W (1866) Mittheilung über Fische (*Protopterus*, *Auliscops*, *Labrax*, *Labracoglossa*, *Nematocentris*, *Serranus*, *Scorpis*, *Opisthognathus*, *Scombresox*, *Acharnes*, *Anguilla*, *Gymnomuraena*, *Chilorhinus*, *Ophichthys*, *Helmichthys*). Monatsbericht der Königlich Preussischen Akademie der Wissenschaften zu Berlin 1866: 509–526.

[B13] Shen S-C Wu K-Y (2011) Fishes of Taiwan. National Museum of Marine Biology and Aquarium, Checheng, 896 pp. [In Chinese]

[B14] Shen S-C Yu L-C Yeh H-S (1986) Additions to the fish-fauna from the adjacent waters around Taiwan (I). Journal of Taiwan Museum 39(1): 65–74.

[B15] Smith-Vaniz WF (1989) Revision of the jawfish genus *Stalix* (Pisces: Opistognathidae), with descriptions of four new species. Proceedings. Academy of Natural Sciences of Philadelphia 141: 375–407.

[B16] Smith-Vaniz WF (1999) Family Opistognathidae. In: Carpenter KE Niem VH (Eds) Species Identification Guide for Fisheries Purposes.The Living Marine Resources of the Western Central Pacific. Bony Fishes, Part 2 (Mugilidae to Carangidae). FAO, Rome, 2588–2589.

[B17] Smith-Vaniz WF (2003) Family Opistognathidae. In: Carpenter KE (Ed.) The Living Marine Resources of the Western Central Atlantic.Volume 3: Bony Fishes, Part 2 (Opistognathidae to Molidae). FAO Species Identification Guide for Fishery Purposes and American Society of Ichthyologist and Herpetologists Special Publication No. 5. FAO, Rome, 1375–1378.

[B18] Smith-Vaniz WF (2009) Three new species of Indo-Pacific jawfishes (*Opistognathus*: Opistognathidae), with the posterior end of the upper jaw produced as a thin flexible lamina. Aqua. International Journal of Ichthyology 15(2): 69–108.

[B19] Smith-Vaniz WF (2016) *Opistognathusensiferus*, a new species of jawfish (Opistognathidae) from the Gulf of Mannar, India, with redescription of *O.solorensis* Bleeker. Zootaxa 4196(2): 278–288. 10.11646/zootaxa.4196.2.6 27988676

[B20] Smith-Vaniz WF (2017) Descriptions of a new genus and two new species of Caribbean deep-water jawfishes (Teleostei: Opistognathidae). Journal of the Ocean Science Foundation 26: 46–58.

[B21] Smith-Vaniz WF (2023) Review of Indo-West Pacific jawfishes (*Opistognathus*: Opistognathidae), with descriptions of 18 new species. Zootaxa 5252(1): 1–180. 10.11646/zootaxa.5252.1.1 37044739

[B22] Smith-Vaniz WF Yoshino T (1985) Review of Japanese jawfishes of the genus *Opistognathus* (Opistognathidae) with description of two new species. Japanese Journal of Ichthyology 32(1): 18–27. 10.11369/jji1950.32.18

[B23] Tashiro S (2019) Family Opistognathidae. In: Koeda K Ho H-C (Eds) Fishes of Southern Taiwan.National Museum of Marine Biology & Aquarium, Checheng, 673–674. [In English and Chinese]

